# Exploring knowledge, attitudes and practice toward medication therapy management services among pharmacists in Yemen

**DOI:** 10.1371/journal.pone.0301417

**Published:** 2024-04-05

**Authors:** Najmaddin A. H. Hatem, Mohamed Izham Mohamed Ibrahim, Seena A. Yousuf

**Affiliations:** 1 Department of Clinical Pharmacy, College of Clinical Pharmacy, Hodeidah University, Al-Hudaydah, Yemen; 2 Department of Clinical Pharmacy and Practice, College of Pharmacy, QU Health, Qatar University, Doha, Qatar; 3 Social Medicine and Public Health Department, Faculty of Medicine and Health Sciences, Aden University, Aden, Yemen; Lahore Medical and Dental College, PAKISTAN

## Abstract

Medication therapy management (MTM) refers to the activities provided by pharmacists that patients recognize as evidence of care being provided. It encompasses the services that patients value and consider valuable. Many developing nations like Yemen have had poor implementation of MTM services. Thus, this research assessed the Knowledge, Attitudes, and Practices (KAP) of Yemen pharmacists regarding MTM. We conducted a cross-sectional study using a self-administered questionnaire among pharmacists in Sana’a, Yemen. They were recruited through convenience sampling. The alpha level of 0.05 was used to determine statistical significance. Four hundred and sixty-one (461) pharmacists completed the questionnaire. About 70% were working in community pharmacies and 57.3% had (1–5) years of experience in pharmacy practice. The younger pharmacists had a higher level of knowledge than pharmacists with older age with median and IQR of 1.2(1.2–1.4) and 1.2(1–1.4) respectively (*p* < 0.001). Yemen pharmacists have positive attitudes toward MTM indicating a moderated level of attitudes with a median and IQR of 3.8(3.5–4). Hospital pharmacists expressed more positive attitudes toward MTM (*P* < 0.001) than pharmacists from other areas of practice. Only 11% of sampled pharmacists frequently offered MTM services. The top MTM service reported by Yemen pharmacists was "Performing or obtaining necessary assessments of the patient’s health status". However, "Formulating a medication treatment plan" received the least provided MTM service among Yemen pharmacists. Even though MTM services are not commonly utilized in pharmacy practice, Yemeni pharmacists have positive attitudes concerning MTM. Efforts are needed to enhance their MTM knowledge and the value of providing MTM services as well as to develop a culture of continuing pharmacy education about MTM among pharmacists.

## Introduction

Medication errors are a worldwide problem. They are "any errors occurring anywhere in the medication use process" [1]. Their incidence rates vary with the nation, but they are most prevalent in individuals who take many medications [2]. According to a study from Yemen, clinical pharmacists gave 2670 interventions or recommendations focused on drug-related problems to the 957 hospital patients who were a part of the study, the majority of which were recognized by approximately 74% in intensive care units [3]. A 42% medication mistake rate was recorded in another study from Sweden [4]. About 6–7% of all inpatients in some countries or regions encountered a medication error, and more than two-thirds of these were preventable [5, 6]. The danger of medication errors must, therefore, be reduced through an efficient mechanism. Given these elevated risks, the Medicare Modernization Act of 2003 mandates creating Medication therapy management (MTM) services for patients with several chronic diseases and/or who are taking multiple medications. MTM is "a service or set of services that optimize therapeutic results for individual patients" [7]. To regulate the patient’s condition, avoid drug-related problems (DRPs), and guarantee the safe and effective use of medications, teamwork between the patient, pharmacist, physician, and other healthcare professionals is required. Evaluating the patient’s medical history and medication profile, improving the patient’s understanding of the disease state and his or her drug therapy, assisting patients in self-monitoring for both desirable and undesirable drug-related effects, and collaborating with other members of the healthcare team to optimize drug therapy are all examples of MTM services [8, 9]. The purpose of these services is to reduce existing or anticipated DRPs to enhance drug utilization. MTM allows pharmacists to engage in the treatment and management of patient cases [10]. As a consequence, they can identify additional DRPs, encourage rational drug usage, and assure medical safety and efficacy, particularly in older adults with comorbidities and multiple medications [3, 11, 12]. Furthermore, expert prescription recommendations offered by pharmacists will raise patients’ understanding of medications, enhance adherence, and lower the financial burden [13–15].

MTM and pharmaceutical care have been used interchangeably, however, they are not the same thing. MTM are the activities that are recognized by the patient as evidence that care is being provided, it is what is recognized as valuable by the patient. However, services that involve decisions that can result in both lifesaving and life-ending consequences cannot be provided without a clearly defined professional mandate and ethics framework that is the foundation of these activities called Pharmaceutical care [5].

Similar medication management strategies have also been adopted in other nations, including the UK’s Medication Use Review (MUR), Australia’s Quality Use of Medicine (QUM), and Residential Medication Management Review (RMMR) [16, 17]. However, the USA MTM approach has been accepted for use in many nations [18, 17]. According to a 2004 survey conducted in the United States, just about 17% of pharmacies provided disease-state management services, and only 10% of pharmacists provided MTM services [19]. However, Alshehri et al.’s study in Saudi Arabia revealed that pharmacists greatly desired to offer MTM services and a positive attitude toward MTM [20]. Despite various barriers, Al-Tameemi et al observed that hospital pharmacists had positive attitudes toward MTM [21]. Jordanian pharmacists came to similar conclusions [22].

Nonetheless, there is a scarcity of data to assess MTM service awareness and implementation in Yemen. This is the first study to evaluate the Knowledge, Attitudes, and Practices (KAP) of pharmacists in Yemen toward MTM service. The results of this study might be used to enhance the MTM, medical services, and clinical pharmacy implementation for patients in hospitals and community pharmacies. A Knowledge, Attitudes, and Practices (KAP) survey can provide valuable insights into pharmacists’ engagement with medication therapy management (MTM) services in Yemen. This survey can assess Yemeni pharmacist’s understanding of MTM services, attitudes towards them, and their engagement in practice, and their ability to deliver patient-centered care. Relevant literature supports the importance of KAP assessments for MTM services, such as studies in Saudi Arabia, Malaysia, and other countries [18, 20, 21]. Identifying educational needs, addressing attitudes and barriers, and enhancing practice patterns are essential for enhancing pharmacists’ knowledge, attitudes, and behaviors. By addressing these factors, targeted interventions can be developed to improve the quality of pharmaceutical care services. Understanding current practice patterns can inform strategies for improving service delivery, patient counseling, and collaboration with other healthcare professionals.

## Material and methods

### Study setting and design

The study used a cross-sectional self-administered questionnaire design, to evaluate the knowledge, attitudes, and practice (KAP) of pharmacists in Yemen toward medication therapy management service. The study took place in Sana’a the capital of Yemen, at more than 100 community pharmacies, more than 10 private hospitals, and three major tertiary public hospitals.

### Study participants and sample size

According to reports, the overall number of pharmacists in Yemen in 2019 was nearly 18000 [23]. As a result, the sample size was calculated using the Raosoft sample size online calculator (http://www.raosoft.com/samplesize.html). Using the collected target population size and assuming a margin of error of 5%, a confidence interval of 95%, and a non-response rate of 20%, the following parameters were included. The minimum final sample size was 453 participants. We invited 500 pharmacists via convenience sampling. All trainee pharmacists, as well as those on leave throughout the research period, were excluded.

### Development of questionnaire

A questionnaire was developed to assess: the pharmacists’ knowledge about MTM, their attitudes toward MTM, and the frequency of offering MTM services. After reviewing the literature on MTM services [21, 22], a structured questionnaire was developed in English than translated to local Arabic to address all of the primary aspects of the study and to apply to the Sana’a, Yemeni population. The definition, components and goals of MTM were represented in the knowledge part using five correct/incorrect items. The attitudes items were measured using 6items on a five-point Likert scale (1 = strongly disagree, 2 = disagree, 3 = neutral, 4 = agree and 5 = strongly agree). The final part assessed the frequency of offering MTM services during pharmacist’s daily practices, and it was measured using 8-items on a five-point Likert scale (1 = Never, 2 = Rarely, 3 = Sometimes, 4 = Most of Times and 5 = All Times). The questionnaire was then examined for suitability and content relevancy by five academics in the field of clinical pharmacy from diverse universities in Yemen. In response to their ideas, minor improvements were made. Face validity was also verified by distributing the questionnaire to ten community pharmacists. Besides, for reliability, a pilot study was done among 10 pharmacists working at different community pharmacies, and they were excluded from the final analysis. Because the pharmacists’ attitude toward MTM was measured using a five-point Likert scale, reliability was assessed with Cronbach’s alpha (0.720). The questionnaire’s final structure was then developed based on every participant’s comments. The survey was introduced in both English and local Arabic.

### Participants consent and ethical approval

Before participating in the study, participants were informed about the purpose, procedures, potential risks, and benefits of the study. They had the opportunity to ask any questions they may have. Verbal consent was obtained from each participant to confirm their voluntary participation. Independent pharmacy students who were not involved in the research, collected data and obtained verbal consent to ensure compliance with ethical standards. The Research and ethics committee at Aden University accepted the study’s design and methodology in accordance with the International Conference on Harmonization (ICH) with research code: (REC-153-2023).

### Data collection

The bilingual paper-based questionnaire was handed out to participants at their pharmacy practice sites in Sana’a, Yemen’s capital city. They were recruited using convenience sampling. Survey responses were gathered and forwarded to the lead researcher. The majority the questionnaires were filled out and returned anonymously. The questionnaire took about 5–10 min to complete. The data collection lasted two weeks later, after taking the ethical approval on 19 June 2023.

### Data analysis

The statistical software for the social sciences (SPSS) version 26 (Armonk, NY: IBM Corp.) was used for coding and analysis, the data was double-checked to minimize data entry errors. To examine the data for normality, the Kolmogorov-Smirnov test was applied. The respondents’ socio-demographic data and items of knowledge, attitudes, and frequency of MTM services provided by pharmacists were all calculated using frequencies and percentages. The median scores and interquartile ranges of the pharmacists’ attitudes were also presented. The Mann-Whitney U test and the Kruskal-Wallis one-way analysis of variance test were used to compare the pharmacists’ socio-demographic data and attitude total score. We applied the Chi-square test to compare the pharmacists’ responses to items of their knowledge and frequency of offering MTM services to their socio-demographic variables.

## Results

### Pharmacists’ characteristics

The study questionnaire was completed by 481 (response rate of 96.2%). Twenty surveys with two or more unanswered questions were eliminated. The total number of usable questionnaires was therefore 461. Most of the respondents (66.4%) were within the age of 20–30 years and were male (63.5%). More than half (57.5%) were bachelor degree holders, were in community pharmacy practice (67.9%) and 57.3% were within 1–5 years of practice year. Further details are shown in Table 1.

**Table 1:** Pharmacists’ characteristics and demographics.

Variables		N (%)
**Age**		
	20–30	306 (66.4)
	> 30	155 (33.6)
**Sex**		
	Male	316 (68.5)
	Female	145 (31.5)
**Marital status**		
	Single	83 (18)
	Married	378 (82)
**Highest degree awarded**		
	Diploma	181 (39.3)
	Bachelor	265 (57.5)
	Pharm.D	10 (2.2)
	Master & PhD	5 (1.1)
**Pharmacy practice setting**		
	Community pharmacy	313 (67.9)
	Hospital pharmacy	79 (17.1)
	Pharmaceutical marketing	69 (15)
**Number of practice years**		
	1–5	264 (57.3)
	6–10	169 (36.7)
	> 10	28 (6.1)

### Knowledge

Table 2 shows the moderate knowledge of respondents towards MTM. About 74.3% had an overall correct understanding of knowledge items regarding MTM. Nearly all the respondents (92.4%) were familiar with the definition and MTM concept. Regarding the five core components of the MTM services, 79% of the respondents were aware of them. However, a good number of pharmacists did not recognize the role of providing MTM services in improving the understanding of medication uses, medication adherence, detection of medication-related problems adherence and disease state management. However, a sizeable percentage of pharmacists did not recognize the role of providing MTM services in improving the understanding of medication uses, medication adherence, detection of medication-related problems adherence and disease state management as seen in incorrect responses to statements 3 (26.3%) and 5 (30.8%) respectively.

**Table 2:** Pharmacist knowledge toward MTM.

Statement	Response, n(%)	
	Correct	Incorrect
MTM is defined as a service or group of services that: optimize therapeutic outcomes for individual patients.	426(92.4%)	35(7.6%)
Core elements of MTM service are Medication Therapy Review (MTR), Personal Medication Record (PMR), Medication Related Action Plan (MAP), Intervention or Referral and Documentation and Follow-Up.	364(79%)	97(21%)
Medication therapy management services have three goals which are: to improve the understanding of medication uses, medication adherence and detection of medication-related problems.	339(73.7%)	121(26.3)
Any patient who uses prescription and nonprescription medications, herbal products or other dietary supplements could potentially benefit from MTM service.	266(57.3%)	195(42.3%)
The primary role of MTM service is aid with adherence and disease state management.	319(69.2%)	142(30.8%)

### Attitudes

Table 3 shows that Yemen pharmacists have positive attitudes toward MTM indicating a moderated level of attitudes with a median and IQR of 3.8(3.5–4). About three quarter (78.8%) of the respondents supported "reviewing patient medication profiles" and "providing interventions besides the traditional dispensing tasks". Besides, 73% agree or strongly agree that "providing MTM services would provide patients with adequate and beneficial information about their chronic disease and medication therapies". However, about 62% believed "Patient’s health outcomes would be improved when medications are monitored by a pharmacist when compared to other health care providers".

**Table 3:** Pharmacist’s attitudes toward MTM.

Statement	Response, n(%)					Median (IQR)
	Strongly Agree	Agree	Neutral	Disagree	Strongly Disagree	
Besides the processes of normal dispensing functions, reviewing patient’s medication profile and providing interventions are important as roles of pharmacist to prevent adverse effects.	29(6.3)	334(72.5)	71(15.4)	13(2.8)	14(3)	4(4–4)
By applying MTM service, patients would receive adequate and beneficial information about their chronic disease (s) and medication therapies from their providers.	30(6.5)	305(66.2)	94(20.4)	24(5.2)	8(1.7)	4(3–4)
By considering the core elements of MTM service, do you agree that MTM service is valuable.	25(5.4)	272(59)	131(28.4)	23(5)	10(2.2)	4(3–4)
Patient’s health outcomes would be improved when medications are monitored by a pharmacist as compared to other health care providers.	39(8.5)	245(53.3)	133(28.9)	31(6.7)	12(26)	4(3–4)
Applying MTM service requires more knowledge than basic information of pharmacy practice.	50(10.9)	266(57.8)	108(23.5)	24(5.2)	12(2.6)	4(3–4)
Providing MTM service is a unique opportunity for pharmacists to participate in patient care at a broader spectrum.	71(15.4)	238(51.6)	106(23)	32(6.9)	14(3)	4(3–4)

### Practice

In Table 4 the overall respondents’ sum of "most of times" and "all times" of offering MTM services was 11% only. The top three Medication Therapy Management (MTM) services reported by Yemeni pharmacists were consistently offered ’most of the time’ and ’all the time,’ as follows: "Performing or obtaining necessary assessments of the patient’s health status", "Providing verbal education and training designed to enhance patient understanding and appropriate use of his/her medications" and "Performing a comprehensive medication review to identify, resolve, and prevent medication-related problems, including adverse drug event" (13.7%, 11.5% and 11.1%) respectively. "Formulating a medication treatment plan" received the least provided MTM service among Yemen pharmacists.

**Table 4:** Frequency of offering MTM services.

MTM service	Response, n(%)				
	All Times	Most of Times	Never	Rarely	Sometimes
Performing or obtaining necessary assessments of the patient’s health status.	17(3.7)	46(10)	92(20)	57(12.4)	249(54)
Formulating a medication treatment plan.	12(2.6)	23(5)	84(18.3)	138(30)	203(44.1)
Selecting, initiating, modifying, or administering medication therapy.	6(1.3)	38(8.2)	128(27.8)	119(25.9)	168(36.5)
Monitoring and evaluating the patient’s response to therapy, including safety and effectiveness.	10(2.2)	36(7.9)	73(15.9)	192(41.9)	147(32.1)
Performing a comprehensive medication review to identify, resolve, and prevent medication-related problems, including adverse drug events.	16(3.5)	35(7.6)	106(23)	159(34.6)	144(31.3)
Documenting the care delivered and communicating essential information to the patient’s other primary care providers.	8(1.7)	34(7.4)	106(23)	165(35.9)	147(32)
Providing verbal education and training designed to enhance patient understanding and appropriate use of their medications.	12(2.6)	41(8.9)	104(22.6)	151(32.8)	153(33.2)
Providing information, support services, and resources designed to enhance patient adherence with his/her therapeutic regimens.	14(3)	34(7.4)	92(20)	148(32.1)	173(37.5)

### Association between demographic variables and pharmacists’ KAP

Table 5 shows the association between demographic variables and pharmacists’ KAP. Except the type of degree obtained, all socio-demographic factors had a statistically significant relationship with the total score of the knowledge. In Table 5, younger pharmacists with median and IQR 1.2(1.2–1.4) had higher levels of knowledge than pharmacists with the age of more than 30 years old (*p* < 0.001). Moreover, pharmacists with fewer years of experience reported a higher level of knowledge than pharmacists with more experience. Further, in the S1 Table, pharmacists with fewer years of experience expressed more knowledge level in two statements "Medication therapy management services have three goals which are: to improve the understanding medication uses, medication adherence and detection of medication-related problems" and "Primary role of MTM service is aid with adherence and disease state management" than pharmacists with more than ten years of experience, [understood by (39.3%) vs. (4.6%) *p* = 0.018 and (37.1%) vs. (5%) *p* = 0.040 respectively].

**Table 5:** The association between demographic variables and pharmacists’ knowledge attitudes and practice.

Variables	Knowledge		Attitudes		Practice	
	Median (IQR)	*P*-value	Median (IQR)	*P*-value	Median (IQR)	*P*-value
**Age** ^A^						
20–30	1.2(1.2–1.4)	<0.001*	3.7(3.33–4)	0.001*	2.4(2–2.62)	0.080
> 30	1.2(1–1.4)		3.8(3.66–4)		2.4(2.1–2.75)	
**Gender** ^A^						
Male	1.2(1.2–1.4)	0.004*	3.7(3.33–4)	0.766	2.4(2–2.62)	0.102
Female	1.2(1–1.4)		3.8(3.66–4)		2.4(2.12–2.62)	
**Marital status** ^A^						
Single	1.2(1.2–1.4)	0.024*	3.7(3.33–3.91)	0.031*	2.4(2.12–2.63)	0.495
Married	1.2(1–1.4)		3.8(3.5–4)		2.4(2–2.63)	
**Highest degree awarded** ^C^						
Diploma	1.2(1.2–1.4)	0.606	3.8(3.5–4)	0.295	2.4(2–2.63)	0.128
Bachelor	1.2(1–1.4)		3.7(3.5–4)		2.4(2–2.63)	
PharmD	1.2(1–1.4)		3.9(3.66–4.16)		2.9(2.25–3.25)	
Master & PhD	1.2(1–1.2)		3.8(3.66–4)		2.4(1.57–2.63)	
**Pharmacy practice setting** ^C^						
Community pharmacy	1.2(1.2–1.4)	<0.001*	3.7(3.33–4)	<0.001*	2.4(2–2.63)	0.023*
Hospital pharmacy	1(1–1.2)		4(3.66–4)		2.5(2.25–2.67)	
Pharmaceutical marketing	1.2(1.2–1.4)		3.7(3.5–3.83)		2.3(2–2.5)	
**Number of practice Years** ^C^						
1–5	1.2(1.2–1.4)	<0.001*	3.7(3.33–4)	0.024*	2.4(2–2.63)	0.070
6–10	1.2(1–1.4)		3.8(3.5–4)		2.4(2–2.7)	
> 10	1.2(1.1–1.4)		3.7(3.5–4)		2.3(1.88–2.4)	

^A^ Mann-Whitney U test,

^C^ Kruskal-Wallis test,

* the value with P< 0.05

Factors including pharmacy practice site, years of practice experience, marital status, and age had a statistically significant relationship with pharmacists’ overall attitudes towards MTM (p < 0.001, p = 0.024, p = 0.031, p = 0.001 respectively). Hospital pharmacists expressed more positive attitudes on four items of the attitudes toward MTM "By applying MTM service, patients would receive adequate and beneficial information about their chronic disease (s) and medication therapies from their providers", "By considering the core elements of MTM service, do you agree that MTM service is valuable", "Patient’s health outcomes would be improved when medications are monitored by a pharmacist as compared to other health care providers" and "Applying MTM service requires more knowledge than basic information of pharmacy practice" (*P =* 0.005, *p =* 0.003, *p =* 0.001, *p =* 0.021 respectively). Further information can be found in S2 Table.

None of the socio-demographic indicators had a significant impact on the overall score of the MTM services practice except for the pharmacy practice setting. Hospital pharmacists expressed less practice scores than other pharmacy practice settings with a median and IQR of 2.5 (2.25–2.67), (*p* = 0.023).

## Discussion

Our research is the first to investigate the Knowledge, Attitudes and Practice (KAP) of pharmacists in Yemen toward medication therapy management service. The study revealed that pharmacists’ knowledge is good despite no real adoption of the MTM services in their daily practice. It is important to point out that our findings reveal that younger pharmacists and pharmacists with fewer years of experience had higher levels of overall knowledge regarding MTM however, we found it the opposite when it comes to overall attitudes scores. The top MTM service reported by Yemeni pharmacists was "Performing or obtaining necessary assessments of the patient’s health status".

The definition of MTM was recognized by a large number of pharmacists in the current study. These findings were superior to those of comparable research done in Malaysia [21], while another study in Saudi Arabia reported more significant results [20]. According to the study findings, pharmacist knowledge of MTM is generally good, however their failure to recognize the two items of the goals in delivering MTM services is inadequate. Because of pharmacists’ drug expertise and patient access, they have been identified as valuable providers of MTM services [7]. The pharmacist’s engagement in MTM services has resulted in increased medication adherence and clinical outcomes, as well as decreased hospitalizations and drug costs [24–27]. Additionally, data gathered from Minnesota’s Fairview Health System found that around 85% of patients had at least one DRP, and more than half of patients’ health conditions improved after obtaining complete medication management [28].

It is essential to point out that our findings reveal that younger pharmacists and pharmacists with fewer (1–5) years of experience had higher levels of overall knowledge regarding MTM. Furthermore, pharmacists with (1–5) years of experience well-recognized the two statements describing the primary goals of delivering MTM services. One possible explanation for these findings is Yemeni pharmacy education’s current shift to a more patient-oriented curriculum, with many universities in recent years beginning to provide more courses that state the new patient-oriented roles, as well as adopting clinical pharmacy education [29, 30].

Despite the fact that MTM and clinical pharmacy services are limited to some pharmacy practice settings, pharmacists show positive attitudes toward providing MTM services with a median and IQR of 3.8(3.5–4). The results are more favorable than those revealed in a comparable study done among Jordanian community pharmacists [22]. Higher findings, however, have been recorded in Malaysia [21]. According to the current study, respondents agreed and strongly agreed that the pharmacist role should include new patient-oriented tasks such as "Medication Therapy Review (MTR), Personal Medication Record (PMR), Medication Related Action Plan (MAP), Intervention or Referral, and Documentation and Follow-Up," and that "providing MTM services is a unique opportunity for pharmacists to participate in patient care", Proactive positive attitudes regarding patient-oriented responsibilities and providing MTM services are necessary from pharmacists if MTM services are to be adopted into regular pharmacy practice settings in Yemen. This outcome was consistent with a prior study conducted among hospital pharmacists in Penang, Malaysia [21]. Approximately 70% of pharmacists feel that "providing MTM services requires more knowledge than basic pharmacy practice knowledge." This was because pharmacy school curricula in Yemen have been increasingly focused on providing patient-centered services over the years. Furthermore, it is likely that practicing pharmacists sought additional training in this field or gained through their practical experience. However, our survey found that around 65% of respondents agreed and strongly agreed that MTM services are helpful, this finding was similar to a previous study done in Jordan [22].

It is worth noting that older pharmacists and those with more years of experience in practice exhibited more positive attitude scores in response to our study. This could be attributed to the fact that pharmacists with extensive experience are familiar with the current practice model, along with its inherent challenges and limitations. On the other hand, recent pharmacy school graduates, who have been prepared for expanded responsibilities in patient care, might encounter frustration due to a disconnect between what they were taught and the realities of practice.

Furthermore, our study revealed a generally higher positive attitude towards Medication Therapy Management (MTM) among hospital pharmacists compared to their counterparts in community pharmacies and pharmaceutical marketing. This observation can be attributed to the broader clinical competencies, duties, and exposure to case studies that hospital pharmacists typically possess. In addition, hospital pharmacists provide more support to physicians and have access to comprehensive patient medical profiles, which further reinforces the value they see in MTM [29]. In addition medical information in Yemen is primarily accessible in hospitals, community pharmacies lack easy access to patient medical profiles. These findings align with the prevailing healthcare landscape in Yemen [30].

In terms of practice, only 11% of pharmacists frequently offered MTM services, they reported a median and IQR of 2.4(2–2.6). This in line with a study done in 2004 a USA-wide survey, where only 10% of pharmacists provided MTM services [19]. In a recent systematic review encompassing 13 studies on the practice of MTM, only five studies reported that pharmacists applied certain elements of MTM [18]. This suggests a notable variation in the application of MTM practices among the studies analyzed. In contrast, a significant number of other studies reported higher levels of application of MTM elements [21, 22]. There are two possible explanations for the current lack of pharmacists providing Medication Therapy Management (MTM) services. First, the dominance of traditional tasks, such as dispensing medication, may be prioritized over providing MTM services [29]. This suggests that pharmacists might not have sufficient knowledge or training to offer comprehensive MTM [29]. Secondly, the increase in the number of prescriptions and the significant number of patients visiting pharmacists may contribute to the shortage of pharmacists providing MTM services. In other words lines between pharmacist activities and the pharmacy technician must be clearly defined in order to provide the pharmacist more time and attention to perform patient-oriented tasks [30].

In the current analysis, the most commonly delivered service was "performing or obtaining a necessary assessment of the patient’s health status", which is in line with a similar earlier study conducted in Jordan [22]. The present study has revealed that respondents seldom "formulate a patient medication treatment plan". An outcome-oriented pharmacotherapy approach is essential for successfully achieving the goals of MTM services. The treatment plan is used to determine with the patient how to properly manage his or her medical condition or diseases using pharmacotherapy, and it comprises all of the efforts required to optimize the drug therapy [5]. Our results show that about 10% of the respondents "all times" or "most of the time" "monitor and evaluate the patient’s response to therapy, including safety and effectiveness" and while only 11% of them were "Performing a comprehensive medication review to identify, resolve, and prevent medication-related problems, including adverse drug events". This is despite substantial evidence supporting its effectiveness in enhancing clinical outcomes, preventing adverse drug reactions, and reducing costs across various medical conditions [12, 24, 28]. Documentation of the care delivered to patients and the ability to communicate effectively is essential for assessing their impact on patient outcomes. In the current study, only 9% always or most of the time offer documentation and communication with other primary care providers. MTM services require good communication and collaboration among healthcare members [5].

### Limitations & strengths

The study’s sample had been limited to Sana’a city. Hence the findings cannot be applied to all pharmacists in the country. Another concern is the social desirability bias: respondents may have given favorable comments to adhere to the more socially accepted viewpoint. Furthermore, because the survey was conducted at a given time, it does not account for any long-term changes in respondents’ knowledge, attitudes, or practice concerning MTM and its services. Despite these limitations, we believe this was the first study in Yemen to evaluate pharmacists’ KAP of MTM. Furthermore, the study had a large sample size, and we believe the results give significant information about how pharmacists in Yemen perceive MTM and its services.

### Study implications on practice

Our study has several implications for clinical pharmacy practice in Yemen. It reveals that, despite the absence of MTM in usual pharmacy practice, Yemeni pharmacists had positive thoughts regarding MTM. To promote the provision of MTM services, collaboration between the Yemeni Medical Council (YMC), healthcare institutes, and pharmacy schools is essential. These institutions play a crucial role in advocating for and supporting the integration of MTM services into pharmacy practice in the nation. The study emphasizes the need for further training and education for practicing pharmacists to perform professional duties. Furthermore, the data results from our study raise interesting questions for future research. It is important to identify and address the impediments that may hinder the provision of MTM services in Yemen. This would involve exploring the barriers and challenges faced by pharmacists and developing strategies to overcome them. Additionally, there is a need to analyze the impact of pharmacist-provided MTM on health outcomes in Yemeni patients. This would provide evidence on the effectiveness and benefits of MTM services in improving patient outcomes and healthcare quality in the country.

### Study recommendations

Efforts to enhance the integration of Medication Therapy Management (MTM) in pharmacy practice necessitate comprehensive measures [31]. Pharmacists stand to benefit significantly from workshops and additional training aimed at deepening their understanding of MTM benefits and honing their practical application skills in real-world pharmacy scenarios. Our study underscores the pivotal role of ongoing training and continuous education for pharmacists, emphasizing the intrinsic link between the seamless incorporation of MTM services into routine practice and the crucial role they fulfill in healthcare.

Advocating for a collaborative approach, our findings highlight the significance of team-based care, calling for the active involvement of nurses, doctors, and pharmacists alike. In the Yemeni context, fostering national cooperation is imperative, involving entities such as the Yemeni Medical Council (YMC), healthcare institutions, healthcare educational institutions, and pharmacy schools. Such collaborative efforts are indispensable for the future advancement of MTM services in the country.

Recognizing the importance of patient-centeredness in pharmacy education, it is recommended to enrich the curricula of undergraduate pharmacy students by incorporating essential elements such as pharmaceutical care, MTM concepts, and clinical knowledge and skills [32]. This step ensures a well-rounded education that aligns with evolving healthcare needs and emphasizes a holistic approach to patient well-being.

Looking forward, future studies should delve into identifying and addressing challenges hindering the seamless provision of MTM services in Yemen. This proactive approach aims to pave the way for a more streamlined and effective implementation of MTM, ultimately improving healthcare outcomes and patient satisfaction in the region.

## Conclusions

Our study assessed the Knowledge, Attitudes and Practice (KAP) of pharmacists in Yemen concerning medication therapy management services. The study revealed that pharmacists’ knowledge is good despite no actual adoption of the MTM services in their daily practice. It is important to point out that our findings reveal that younger pharmacists and those with less experience had higher knowledge of MTM. In comparison, older pharmacists and those with more experience had more positive attitudes scores. About 11% of respondents frequently offered MTM services, the top MTM service reported was "Performing or obtaining necessary assessments of the patient’s health status". While, "Formulating a medication treatment plan" received the least provided MTM service among them. Actions are required to improve pharmacists’ MTM understanding and the value of delivering MTM services, as well as establish a culture of continual pharmacy education about MTM among Yemen pharmacists.

## Supporting information

S1 File The bilingual paper-based questionnaire. (PDF)

S1 Table Significant relationship between items of pharmacists’ knowledge towards MTM and socio-demographic characteristics categories. (PDF)

S2 Table Significant relationship between items of pharmacists’ attitudes towards MTM and socio-demographic characteristics categories. (PDF)

S3 Table Significant relationship between items of pharmacists’ practice towards MTM services and socio-demographic characteristics categories. (PDF)
