# Supplementary material for: Exploring knowledge, attitudes and practice toward medication therapy management services among pharmacists in Yemen
Source: PLoS One. 2024 Apr 5;19(4):e0301417. doi: 10.1371/journal.pone.0301417 (PMC10997124; doi:10.1371/journal.pone.0301417)
Supplement: S1 File — (PDF) [file pone.0301417.s001.pdf]

Exploring Knowledge, Attitudes and Practice Toward Medication Therapy Management Services among pharmacists in Yemen

Dear Pharmacist,

Greeting

We are conduction a survey regarding pharmacists knowledge , attitudes and practice toward Medication Therapy Management Services.

We invited you to complete this 10-minute questionnaire and your response are of a great value.

We can assure you that personal information would be confidential and the responses we are going to obtain will be used only for research purposes

Thank you very much for your participation in this study.

Part One: Demographics

Age : …….. 20 - 30 …….. > 30

Gender : …….. Male …….. Female

Marital status : …….. Single …….. Married

Highest degree awarded : ….. Diploma ….. Bachelor ….. Pharm.D ….. Master & PhD

Pharmacy practice setting : ….. Community pharmacy ….. Hospital pharmacy

….. Pharmaceutical marketing

Number of practice Years : ….. 1 – 5 ….. 6 – 10 ….. > 10

Part Two : Pharmacist's Knowledge toward medication therapy management

Statement	Correct	Incorrect
MTM is defined as service or group of services that: optimize therapeutic outcomes for individual patients.		
Core elements of MTM service are Medication Therapy Review (MTR), Personal Medication Record (PMR), Medication Related Action Plan (MAP), Intervention or Referral and Documentation and Follow-Up.		
Medication therapy management services have three goals which are: to improve the understanding medication uses, medication adherence and detection of medication related problems.		
Any patient who uses prescription and nonprescription medications, herbal products or other dietary supplements could potentially benefit from MTM service.		
Primary role of MTM service is aid with adherence and disease state management.		

Part Three : Pharmacist's attitudes toward medication therapy management

Statement	Strongly Agree	Agree	Neutral	Disagree	Strongly Disagree
Besides the processes of normal dispensing functions, reviewing patient's medication profile and providing interventions are important as roles of pharmacist to prevent adverse effects.					
By applying MTM service, patients would receive adequate and beneficial information about their chronic disease (s) and medication therapies from their providers.					
By considering the core elements of MTM service, do you agree that MTM service is valuable.					
Patient's health outcomes would be improved when medications are monitored by a pharmacist as compared to other health care providers.					
Applying MTM service requires more knowledge than basic information of pharmacy practice.					
Providing MTM service is a unique opportunity for pharmacists to participate in patient care at a broader spectrum.					

Part Four : Medication therapy management services practice

How Frequently you offer these MTM services?	All Times	Most of Times	Never	Rarely	Sometimes
Performing or obtaining necessary assessments of the patient's health status.					
Formulating a medication treatment plan.					
Selecting, initiating, modifying, or administering medication therapy.					
Monitoring and evaluating the patient's response to therapy, including safety and effectiveness.					
Performing a comprehensive medication review to identify, resolve, and prevent medication-related problems, including adverse drug events..					
Documenting the care delivered and communicating essential information to the patient's other primary care providers.					
Providing verbal education and training designed to enhance patient understanding and appropriate use of his/her medications.					
Providing information, support services, and resources designed to enhance patient adherence with his/her therapeutic regimens.					

Thanks,,,
