# Supplementary material for: Exploring knowledge, attitudes and practice toward medication therapy management services among pharmacists in Yemen
Source: PLoS One. 2024 Apr 5;19(4):e0301417. doi: 10.1371/journal.pone.0301417 (PMC10997124; doi:10.1371/journal.pone.0301417)
Supplement: S1 Table — (PDF) [file pone.0301417.s002.pdf]

S1 Table. Significant relationship between items of pharmacists' knowledge towards MTM and socio-demographic characteristics categories.

Statement ***VS*** **Variable** [B]	**Category**	**Correct Answer**	***P*-Value**
Core elements of MTM service are Medication Therapy Review (MTR), Personal Medication Record (PMR), Medication Related Action Plan (MAP), Intervention or Referral and Documentation and Follow-Up.* **Pharmacy practice setting**	Community pharmacy<br>Hospital pharmacy<br>Pharmaceutical marketing<br>Total	51.2%<br>15.4%<br>12.4%<br>79%	0.014
Medication therapy management services have three goals which are: to improve the understanding medication uses, medication adherence and detection of medication related problems.* **Age**	20 - 30<br>> 30<br>Total	46.5%<br>27.2%<br>73.7%	0.016
Medication therapy management services have three goals which are: to improve the understanding medication uses, medication adherence and detection of medication related problems.* **Number of practice Years**	1 – 5<br>6 – 10<br>> 10<br>Total	39.3%<br>29.8%<br>4.6%<br>73.7%	0.018
Any patient who uses prescription and nonprescription medications, herbal products or other dietary supplements could potentially benefit from MTM service.* **Pharmacy practice setting**	Community pharmacy<br>Hospital pharmacy<br>Pharmaceutical marketing<br>Total	31%<br>3.9%<br>7.4%<br>42.3%	0.001
Primary role of MTM service is aid with adherence and disease state management.* **Marital status**	Single<br>Married<br>Total	58.7%<br>10.6%<br>69.2%	0.027
Primary role of MTM service is aid with adherence and disease state management.* **Number of practice Years**	1 – 5<br>6 – 10<br>> 10<br>Total	37.1%<br>27.1%<br>5%<br>69.2%	0.040

[B] Chi square test, * Vs, Significance ($p < 0.05$)
