# Supplementary material for: Exploring knowledge, attitudes and practice toward medication therapy management services among pharmacists in Yemen
Source: PLoS One. 2024 Apr 5;19(4):e0301417. doi: 10.1371/journal.pone.0301417 (PMC10997124; doi:10.1371/journal.pone.0301417)
Supplement: S2 Table — (PDF) [file pone.0301417.s003.pdf]

**S2 Table. Significant relationship between items of pharmacists' attitudes towards MTM and socio-demographic characteristics categories.**

| Statement ***VS*** **Variable** | **Category** | **Median (IQR)** | ***P*-value** |
|---|---|---|---|
| Besides the processes of normal dispensing functions, reviewing patient's medication profile and providing interventions are important as roles of pharmacist to prevent adverse effects. * **Marital status** [A] | Single<br>Married | 4(3 – 4)<br>4(4 – 4) | 0.049 |
| Besides the processes of normal dispensing functions, reviewing patient's medication profile and providing interventions are important as roles of pharmacist to prevent adverse effects. * **Number of practice Years** [C] | 1 – 5<br>6 – 10<br>> 10 | 4(3 – 4)<br>4(4 – 4)<br>4(3 – 4) | 0.036 |
| By applying MTM service, patients would receive adequate and beneficial information about their chronic disease (s) and medication therapies from their providers. * **Age** [A] | 20 - 30<br>> 30 | 4(3 – 4)<br>4(4 – 4) | <0.001 |
| By applying MTM service, patients would receive adequate and beneficial information about their chronic disease (s) and medication therapies from their providers. .* **Marital status** [A] | Single<br>Married | 4(3 – 4)<br>4(3 – 4) | 0.017 |
| By applying MTM service, patients would receive adequate and beneficial information about their chronic disease (s) and medication therapies from their providers***** **Pharmacy practice setting** [C] | Community pharmacy<br>Hospital pharmacy<br>Pharmaceutical marketing | 4(3 – 4)<br>4(4 – 4)<br>4(3 – 4) | 0.005 |
| By applying MTM service, patients would receive adequate and beneficial information about their chronic disease (s) and medication therapies from their providers. * **Highest degree awarded** [C] | Diploma<br>Bachelor<br>PharmD<br>Master & PhD | 4(3 – 4)<br>4(3 – 4)<br>4(4 – 5)<br>4(4 – 4) | 0.008 |

| | | | |
|---|---|---|---|
| By considering the core elements of MTM service, do you agree that MTM service is valuable. * **Age** [A] | 20 - 30<br>> 30 | 4(3 – 4)<br>4(3 – 4) | <0.001 |
| By considering the core elements of MTM service, do you agree that MTM service is valuable. * **Gender** [A] | Male<br>Female | 4(3 – 4)<br>4(3 – 4) | 0.034 |
| By considering the core elements of MTM service, do you agree that MTM service is valuable.* **Number of practice Years** [C] | 1 – 5<br>6 – 10<br>> 10 | 4(3 – 4)<br>4(3 – 4)<br>4(3 – 4) | 0.030 |
| By considering the core elements of MTM service, do you agree that MTM service is valuable. * **Pharmacy practice setting** [C] | Community pharmacy<br>Hospital pharmacy<br>Pharmaceutical marketing | 4(3 – 4)<br>4(4 – 4)<br>4(3 – 4) | 0.003 |
| Patient's health outcomes would be improved when medications are monitored by a pharmacist as compared to other health care providers. * **Pharmacy practice setting** [C] | Community pharmacy<br>Hospital pharmacy<br>Pharmaceutical marketing | 4(3 – 4)<br>4(4 – 4)<br>4(3 – 4) | 0.001 |
| Applying MTM service requires more knowledge than basic information of pharmacy practice. * **Age** [A] | 20 - 30<br>> 30 | 4(3 – 4)<br>4(4 – 4) | 0.003 |
| Applying MTM service requires more knowledge than basic information of pharmacy practice. * **Marital status** [A] | Single<br>Married | 4(3 – 4)<br>4(3 – 4) | 0.042 |
| Applying MTM service requires more knowledge than basic information of pharmacy practice. * **Pharmacy practice setting** [C] | Community pharmacy<br>Hospital pharmacy<br>Pharmaceutical marketing | 4(3 – 4)<br>4(4 – 4)<br>4(3 – 4) | 0.021 |
| Providing MTM service is a unique opportunity for pharmacists to participate in patient care at a broader spectrum. * **Age** [A] | 20 - 30<br>> 30 | 4(3 – 4)<br>4(4 – 4) | 0.002 |

[A] Mann-Whitney U test, [C] Kruskal-Wallis test, * Vs, Significance ($p < 0.05$)
