# Supplementary material for: Exploring knowledge, attitudes and practice toward medication therapy management services among pharmacists in Yemen
Source: PLoS One. 2024 Apr 5;19(4):e0301417. doi: 10.1371/journal.pone.0301417 (PMC10997124; doi:10.1371/journal.pone.0301417)
Supplement: S3 Table — (PDF) [file pone.0301417.s004.pdf]

S3 Table. Significant relationship between items of pharmacists' practice towards MTM services and socio-demographic characteristics categories.

MTM service ***VS*** **Variables** [B]	Category	All Times	Most of Times	Never	Rarely	Sometimes	*P*-Value
Performing or obtaining necessary assessments of the patient's health status. * **Highest degree awarded**	Diploma<br>Bachelor<br>PharmD<br>Master & PhD	8(1.7)<br>7(1.5)<br>2(0.4)<br>0	19(4.1)<br>22(4.8)<br>5(1.1)<br>0	42(9.1)<br>50(10.8)<br>0<br>0	27(5.9)<br>29(6.3)<br>1(0.22)<br>0	85(18.4)<br>157(34.1)<br>2(0.43)<br>5(1.1)	<0.001
Performing or obtaining necessary assessments of the patient's health status. * **Pharmacy practice setting**	Community pharmacy<br>Hospital pharmacy<br>Pharmaceutical marketing	11(2.4)<br>2(0.5)<br>4(0.9)	36(7.8)<br>4(0.9)<br>6(1.3)	67(14.5)<br>6(1.3)<br>19(4.1)	37(8)<br>12(2.6)<br>8(1.7)	162(35.1)<br>55(11.9)<br>32(6.9)	0.025
Formulating a medication treatment plan.* **Marital status**	Single<br>Married	4(0.9)<br>8(1.7)	4(0.9)<br>19(4.1)	13(2.8)<br>71(15.4)	38(8.3)<br>100(21.8)	23(5)<br>180(39.1)	0.002
Selecting, initiating, modifying, or administering medication therapy.* **Marital status**	Single<br>Married	4(0.9)<br>2(0.4)	9(2)<br>29(6.3)	26(5.7)<br>102(22.2)	19(4.1)<br>100(21.8)	24(5.2)<br>144(31.3)	0.022
Performing a comprehensive medication review to identify, resolve, and prevent medication-related problems, including adverse drug events.* **Number of practice Years**	1 – 5<br>6 – 10<br>> 10	4(0.9)<br>11(2.4)<br>1(0.2)	20(4.3)<br>11(2.4)<br>4(0.8)	56(12.2)<br>39(8.5)<br>11(2.4)	98(21.3)<br>57(12.4)<br>4(0.9)	85(18.5)<br>51(11.1)<br>8(1.7)	0.030
Documenting the care delivered and communicating essential information to the patient's other primary care providers.* **Highest degree awarded**	Diploma<br>Bachelor<br>PharmD<br>Master & PhD	6(1.3)<br>2(0.4)<br>0<br>0	16(3.5)<br>15(3.3)<br>3(0.7)<br>0	37(8)<br>67(14.6)<br>0<br>2(0.4)	70(15.2)<br>92(20)<br>3(0.3)<br>0	52(11.3)<br>88(19.1)<br>4(0.9)<br>3(0.3)	0.040

[B] Chi square test, * Vs, Significance ($p < 0.05$)
